# 

**Published:** 1917-04-28

**Authors:** 


Miss Isabel Briscoe, of Codsall, left £500 each to the Wolverhampton and Staffordshire General Hospital and the Wolverhampton and District Hospital for Women, £100 each to the London Hospital and the Brompton Consumption Hospital, and £50 to the Wolverhampton District Branch of the Queen Victoria Nursing Association.

### Matrons' and Sisters' Appointments.

Victoria Cottage Hospital, Guernsey.—Miss Florence E. Forsdick has been appointed matron. She was at the Hove Sanatorium, Portslade, and then trained for three years at Bradford Royal Infirmary, where, after eighteen months spent in private nursing, she was for seven years sister and assistant matron.

Harton Poor-Law Institution, South Shields.—Miss Martha Jane Horner has been appointed superintendent. She was trained at St. Pancras Infirmary, and has been ward and night sister at Isleworth Infirmary, night sister and temporary assistant matron at Edmonton Infirmary, and home sister and second assistant matron at Shoreditch Infirmary.

Bethnal Green Military Hospital.—Mrs. Christina Hynes (*née* Shepherd) has been appointed sister. She was trained at St. Marylebone Infirmary, and has been ward and home sister at the Policlinico, Rome; matron of Tring Fever Hospital, and ward sister at St. Pancras (South) Infirmary.

Coventry and Warwickshire Hospital.—Miss L. Cowley has been appointed sister. She was trained at the Northampton General Hospital, and has since been day and night sister at the Military Hospital, Weston Favell, Northampton, and night sister at the Warrington Infirmary and Dispensary.

Darlington General Hospital.—Mrs. Nixon has been appointed night sister. She was trained at Essex County Hospital, where she has taken sister's holiday duties. She has been staff nurse, sister, and night superintendent at the City Fever Hospital, Leeds.

Prescot Union Infirmary.—Miss L. G. Stubley has been appointed night sister. She was trained at the Prescot Union Infirmary, where she has since been sister of the medical and surgical wards.

Tunbridge Wells General Hospital.—Miss Edith Annie Hague has been appointed night sister. She was trained at Preston Royal Infirmary, where she has since been temporary night sister and sister of the children's ward.

**NOTE.—Particulars of Appointments for insertion in this column will be welcomed.**

### Gifts and Bequests.

Mr. William Cumming, of Ebor Mount, Huddersfield, woollen merchant, left £100 to the Huddersfield Infirmary.

Mr. W. H. Thorold, of Eaton, Norfolk, left £100 each to St. Bartholomew's Hospital and the Norfolk and Norwich Hospital.

Messrs. James Buchanan and Co., Ltd., and Messrs. John Dewar and Sons, Ltd., have given £500 each to the London Hospital.

Mr. F. J. Mankiewicz, a brother of Lady Wernher, left a number of legacies of £100 each to charities, including Charing Cross Hospital.

Mr. H. C. White, of Sutton Coldfield, left several legacies to local charities, including £1,000 to the Nurses' Home and £50 to the dispensary at Sutton.

Mr. Joseph Taylor, of Aldridge, left the following charitable legacies, amongst others: The Walsall and District Hospital, £20,000; the Walsall Victoria Nursing Institution, £10,000; the Queen's Hospital, Birmingham, £5,000; the Birmingham and Midland Eye Hospital, £2,000; the Wolverhampton and Midland Counties Eye Infirmary, £2,000.

### The Book Market.

LIST OF NEW BOOKS RECEIVED FROM THE FOLLOWING PUBLISHERS:—

H. K. Lewis and Co. : "The Causation of Sex in Man." By E. Rumley Dawson, L.R.C.P. 7s. 6d. net.

Methuen and Co., Ltd. : "Outposts of Mercy." By W. Lucas. 1s. net.

The Scientific Press, Ltd. : "The Expectant Mother." By C. L. Norman. 2d. net. "The Pocket Anatomical Atlas." By a London M.D. 1s. 3d. net.

### Medical Appointments.

University of Sheffield.—Dr. Lucy Naish has been appointed Lady Tutor in Anatomy, *vice* Dr. Sophia Witts, resigned.

### Passing Events and Topics.

### The Hospital for Epilepsy and Paralysis, Maida Vale.

The branch hospital of 100 beds for the treatment and re-education of invalided soldiers suffering from neurasthenia, shell shock, and grave nervous breakdown generally will be opened shortly.

### Motor Ambulance Given to Red Cross.

A scheme initiated in the Hawarden district last November for the provision of a fully-equipped motor ambulance for service abroad has proved successful, having resulted in a collection of £679.

### Death of a Hospital Assistant Secretary.

We regret to record the death of Mr. A. C. Davis, for twenty-three years assistant secretary to the National Hospital for the Paralysed and Epileptic, Queen Square, W.C. Mr. Davis died suddenly, whilst on duty, on the 20th instant.

### Epsom Red Cross Week.

The net receipts of Epsom and District Red Cross week amounted to £6,615 12s. 4d., and a cheque for this amount was handed to the representatives of the British Red Cross Society and the Farmers' Red Cross Fund at a meeting held last week.

RATES OF SUBSCRIPTION: Twelve months, 6/6; Six months, 4/-; Three months, 2/-; post free to any address in the United Kingdom. Abroad, Twelve months, 8/8; Six months, 5/-; Three months, 2/6, post free. THE HOSPITAL "Index" to Volume LX., April to September 1916, is now ready, price 2½d. per copy, post free. Address The Manager, "THE HOSPITAL," The Hospital Building, 28/29 Southampton Street, Strand, London, W.C. 2.

